# Instauración de anticoagulación en fibrilación auricular subclínica detectada por dispositivo: ¿en qué desenlaces impacta?

**DOI:** 10.47487/apcyccv.v5i2.365.

**Published:** 2024-06-24

**Authors:** Hugo Fernando Fuentes Blanco, Diego Alejandro Malagón Albarracín, Francisco Javier González Perdomo, Michael Ortega Sierra

**Affiliations:** 1 Departamento de Medicina, Universidad Del Magdalena, Santa Marta, Colombia. Universidad del Magdalena Departamento de Medicina Universidad Del Magdalena Santa Marta Colombia; 2 Departamento de Medicina, Universidad Militar Nueva Granada, Bogotá, Colombia. Universidad Militar Nueva Granada Departamento de Medicina Universidad Militar Nueva Granada Bogotá Colombia; 3 Departamento de Medicina, Universidad Nacional de Colombia, Bogotá, Colombia. Universidad Nacional de Colombia Departamento de Medicina Universidad Nacional de Colombia Bogotá Colombia; 4 Departamento de Neurocirugía, Universidad Centroccidental Lisandro Alvarado, Barquisimeto, Venezuela. Universidad Centroccidental Lisandro Alvarado Departamento de Neurocirugía Universidad Centroccidental Lisandro Alvarado Barquisimeto Venezuela; 5 Hospital Central Antonio María Pineda, Barquisimeto, Venezuela. Hospital Central Antonio María Pineda Barquisimeto Venezuela

*Sr. Editor.*

La fibrilación auricular subclínica es una entidad de creciente interés en cardiología clínica y electrofisiología que consiste en una fibrilación auricular asintomática y cuyo diagnóstico se realiza a partir del registro electrocardiográfico en un paciente portador de algún tipo de dispositivo intraauricular ^1^. A diferencia de la fibrilación auricular sintomática y debido a su hallazgo incidental con el uso de dispositivos cardiacos, su manejo es incierto ^1^. Uno de los dilemas actuales en el control del riesgo de desenlaces cardiovasculares es decidir si se instaura o no anticoagulación en estos casos, que se conocen como fibrilación auricular subclínica detectada por dispositivos (FASDD), principalmente para disminuir el riesgo de ataque cerebrovascular isquémico ^2^^,^^3^. Considerando que, con el avance de las ciencias biomédicas, será cada vez más accesible al uso de dispositivos inteligentes que podrían ayudar a identificar este tipo de condiciones, se esperaría, eventualmente, una mayor frecuencia de diagnósticos de estos casos y, por lo tanto, mayores retos en la toma de decisiones en la práctica clínica.

Recientemente, un metaanálisis que combinó los resultados de los ensayos NOAH-AFNET 6 y ARTESIA buscó evaluar la eficacia y seguridad de la anticoagulación en la FASDD ^3^. Los investigadores incluyeron en el análisis poco más de 6500 sujetos, a quienes se les administraba apixaban o edoxaban para el manejo de FASDD o para episodios auriculares de alta frecuencia. Los autores identificaron que el uso de anticoagulación redujo el riesgo de un evento cerebrovascular hasta en un 32% (IC 95%: 0,50 - 0,92). Así mismo, la anticoagulación redujo hasta en un 15% el riesgo de muerte cardiovascular, infarto agudo de miocardio, tromboembolismo pulmonar o ictus isquémico por cualquier causa (IC 95%: 0,73 - 1). No obstante, se observó un aumento del 62% en el riesgo de sangrado mayor (IC 95%: 1,05 - 2,51) ^3^. Así, los autores concluyeron que, aunque los datos provenientes de esos ensayos constituyen un elevado nivel de evidencia, soportando la potencial eficacia de la anticoagulación en estos casos para la reducción de diversos desenlaces cardiovasculares, la seguridad debe personalizarse, esencialmente por el riesgo de sangrado mayor ^3^.

Estas mismas conclusiones fueron discutidas por Boriani *et al*. ^2^, quienes apoyan el uso de la anticoagulación, informando previamente del riesgo inherente de sangrado como efecto adverso. Patel & Ruff ^4^ sugieren que sopesar el balance beneficio/riesgo aún es muy crítico, toda vez que debería contarse con evidencia un poco más robusta. Una posición de la Asociación Americana del Corazón consiste en que el abordaje debe instaurarse de forma individual, ya que se desconocen los desenlaces a largo plazo y, específicamente, en otras poblaciones que compartan características que puedan influir directamente en estos estimados ^5^.

Lo que es un hecho, es la relevancia y pertinencia del estudio de esta condición, para tener la oportunidad de reducir el riesgo de desenlaces cardio y cerebrovasculares en aquellos con fibrilación auricular asintomática, reducir costos en salud y disminuir la carga de enfermedad que genera la arritmia más frecuente del mundo ^6^. Desafortunadamente, la evidencia encontrada en la literatura sobre esta condición es heterogénea y proviene esencialmente de población americana y europea ^7^. Por lo tanto, constituye un nicho de interés en investigación e innovación en Latinoamérica. Hace poco, Batista Mendoza *et al*. ^8^ realizaron un análisis bibliométrico sobre la investigación en falla cardiaca donde se observó que no existe tendencia alguna sobre la FASDD, lo que confirma la hipótesis sobre la necesidad de impulsar investigación actual en el tema ^8^.

En conclusión, la anticoagulación en la FASDD podría generar beneficios importantes en la reducción del riesgo de ictus cerebral isquémico, así como otros desenlaces cardiovasculares. Sin embargo, existe un importante riesgo de sangrado mayor, por lo que el abordaje instaurado debe ser personalizado.

## Financiamiento:

Autofinanciado
